# Radiolabeled Antibodies for Cancer Imaging and Therapy

**DOI:** 10.3390/cancers14061454

**Published:** 2022-03-11

**Authors:** Sagun Parakh, Sze Ting Lee, Hui K. Gan, Andrew M. Scott

**Affiliations:** 1 Department of Medical Oncology, Heidelberg, VIC 3084, Australia; sagun.parakh@onjcri.org.au (S.P.); hui.gan@onjcri.org.au (H.K.G.); 2 Olivia Newton-John Cancer Research Institute, Heidelberg, VIC 3084, Australia; szeting.lee@austin.org.au; 3 School of Cancer Medicine, La Trobe University, Heidelberg, VIC 3086, Australia; 4 Department of Molecular Imaging and Therapy, Austin Health, Heidelberg, VIC 3084, Australia; 5 Department of Medicine, University of Melbourne, Heidelberg, VIC 3010, Australia

**Keywords:** radioimmunotherapy, radioisotopes, radiolabeled monoclonal antibodies, theranostics

## Abstract

**Simple Summary:**

Monoclonal antibodies (mAbs) have the ability to specifically target tumor-cell antigens. This unique property has led to their use in the delivery of radioisotopes to tumor sites (scintigraphic imaging and radioimmunotherapy (RIT)). The choice of the radionuclide depends on its unique physical properties and intended use. Using radiolabeled mAbs with imaging techniques provides critical data that are essential for predicting side effects and determining an optimal antibody dose and treatment schedule. While RIT has been successful in the management of hematological malignancies, the treatment of solid tumors remains challenging. Various strategies are being investigated to improve the efficacy of RIT in solid tumors.

**Abstract:**

Radioimmunoconjugates consist of a monoclonal antibody (mAb) linked to a radionuclide. Radioimmunoconjugates as theranostics tools have been in development with success, particularly in hematological malignancies, leading to approval by the US Food and Drug Administration (FDA) for the treatment of non-Hodgkin’s lymphoma. Radioimmunotherapy (RIT) allows for reduced toxicity compared to conventional radiation therapy and enhances the efficacy of mAbs. In addition, using radiolabeled mAbs with imaging methods provides critical information on the pharmacokinetics and pharmacodynamics of therapeutic agents with direct relevance to the optimization of the dose and dosing schedule, real-time antigen quantitation, antigen heterogeneity, and dynamic antigen changes. All of these parameters are critical in predicting treatment responses and identifying patients who are most likely to benefit from treatment. Historically, RITs have been less effective in solid tumors; however, several strategies are being investigated to improve their therapeutic index, including targeting patients with minimal disease burden; using pre-targeting strategies, newer radionuclides, and improved labeling techniques; and using combined modalities and locoregional application. This review provides an overview of the radiolabeled intact antibodies currently in clinical use and those in development.

## 1. Introduction

Since the initial concept of “magic bullets” was proposed over a century ago, through to the discovery of hybridoma technology, monoclonal antibodies (mAbs) are now a vital component in the armamentarium for the management of cancers. The unique ability of mAbs to specifically target a broad variety of tumor-specific antigens has led to their expanded application as antibody-conjugated therapies (ACTs). ACTs combine the specificity of mAbs or antibody fragments, with highly potent payloads often resulting in superior efficacy and/or reduced toxicity [1]. Radioimmunoconjugates (radiolabeled antibodies) are mAb linked to a radionuclide [2]. Radioimmunoconjugates as therapeutic and/or diagnostic agents in the management of cancer have been in development with some success for a few decades now. Significant strides have been made since the first radioimmunoconjugate was developed, leading to improved therapeutic efficacy [3,4]. Mabs and antibody-related therapies can be efficiently labeled with a variety of radionuclides for theranostic purposes. The radionuclides commonly used include actinium-225 (^225^Ac), astatine-211 (^211^At), bismuth-213 (^213^Bi), indium-111 (^111^In), iodine-123 (^123^I), iodine-124 (^124^I), iodine-131 (^131^I), lead-212 (^212^Pb), lutetium-177 (^177^Lu), technetium-99m (^99m^Tc), copper-64 (^64^Cu), gallium-68 (^68^Ga), yttrium-86 (^86^Y), yttrium-90 (^90^Y), and zirconium-89 (^89^Zr) [5]. Based on their radiation properties, therapeutic radionuclides can be classified as β-particles, α-particles, or Auger electron emitters. β- particles are negatively charged electrons emitted from the nucleus with a long range and low linear energy transfer (LET). They are the most frequently used emission type for RIT agents and include lutetium-177(^177^Lu), yttrium-90 (^90^Y), and iodine-131 ^(131^I). Alpha-particles, in contrast, have significantly higher energies, very short path lengths, and high LET. Alpha particles are emerging as an exciting new class of radionuclides with increased biological killing efficacy and lack of non-specific bystander effects seen with β-particle irradiation on normal tissue. These include astatine-211 (^211^At), actinium-225 (^225^Ac), thorium-227 (^227^Th), and bismuth-213 (^213^Bi).

This review provides an overview of radiolabeled intact antibodies currently in clinical use for the detection and treatment of hematological cancers and solid tumors, as well as those in development; examples of such clinical trials are shown in Table 1. We do not discuss smaller engineered antibody-based proteins or peptides, as this is beyond the scope of this review. 

## 2. Molecular Imaging

The ability of mAbs and antibody-related therapeutics to specifically target tumor-specific antigens allows expanding their application for theranostic approaches. Using radiolabeled mAbs with imaging methods, such as single-photon emission computerized tomography (SPECT) or positron-emission tomography (PET), provides vital insights into the pharmacokinetics and pharmacodynamics of therapeutic agents, heterogeneity, and dynamic changes of antigen expression, antigen engagement and uptake in tumors [6]. All of these parameters are critical for guiding and monitoring therapy responses, predicting toxicity, and identifying patients most likely to benefit from treatment. In addition, they provide critical information with direct relevance to the optimization of dosing and scheduling of mAbs. Zirconium-89 (t1/2, 3.27 days) and indium-111 (t1/2, 2.8 days) are two of the most commonly used radionuclides for PET and SPECT imaging, respectively.

### 2.1. Epidermal Growth Factor Receptor (EGFR)

Cetuximab, an IgG1 chimeric EGFR targeting mAb and radiolabeled with zirconium-89, has shown to be well tolerated [7] and demonstrated a strong correlation between uptake and response to cetuximab in patients with mCRC [8]. A larger study by the same group, however, showed no relationship between tumor uptake of ^89^Zr-cetuximab and treatment response [9]. These data suggest, in addition to EGFR expression, that ^89^Zr-cetuximab tumor uptake is influenced by other pharmacokinetic and dynamic mechanisms [9,10]. 

Panitumumab is a humanized anti-EGFR mAb. Panitumumab radiolabeled with a variety of radionuclides, including yttrium-86, copper-64, indium-111, and zirconium-89, which have been evaluated as PET probes for imaging EGFR [11]. Compared to ^86^Y-labeled-cetuximab, ^86^Y-labeled-panitumumab demonstrated higher tumor uptake and significantly lower liver uptake in a malignant mesothelioma model [12]. While ^89^Zr-panitumumab uptake highly correlated with EGFR expression, it is unable to detect EGFR mutations and mutations in downstream signaling pathways, such as KRAS and PTEN [11,13]. A first in human dosimetry study of ^89^Zr-panitumumab in patients with metastatic CRC reported the whole-body effective dose between 0.264 mSv/MBq (0.97 rem/mCi) and 0.330 mSv/MBq (1.22 rem/mCi), with the liver receiving the highest dose [14]. A bio-imaging study evaluating ^89^Zr-Panitumumab in patients with newly diagnosed colon cancer with lymph node involvement is currently enrolling (NCT03764137). 

Nimotuzumab is an affinity-optimized anti-EGFR mAb. Preclinical studies in breast and colorectal carcinoma models demonstrated tumor uptake of ^89^Zr-nimotuzumab increasing up to 168 h post-injection, and, notably, EGFR expression was clearly visualized as early as 24 h post-injection [15]. ^89^Zr-nimotuzumab is being evaluated in a phase I/II trial to demonstrate the feasibility of identifying patients with EGFR-expressing tumors most likely to respond to anti-EGFR therapies (NCT04235114). 

**Figure 1:**
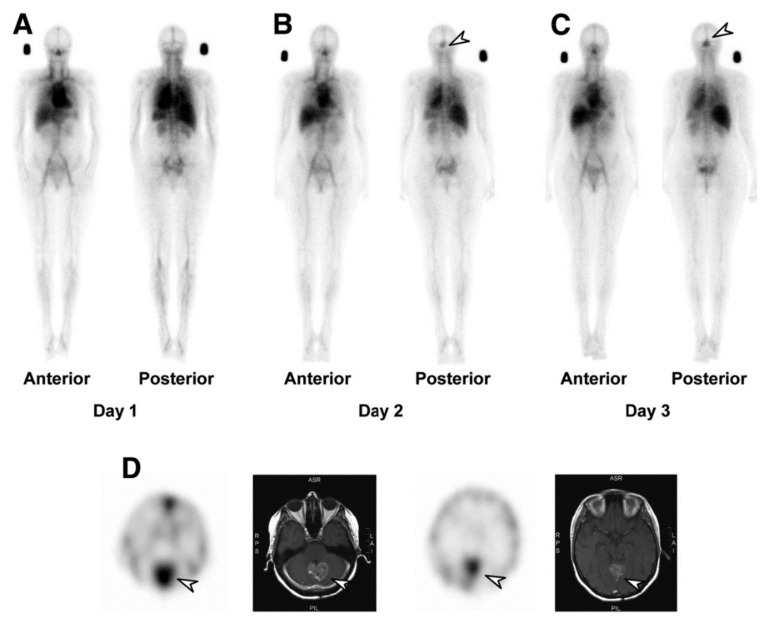
^111^In-ABT-806 (ABT-806i) biodistribution and SPECT/CT images of patient with high-grade glioma on (**A**) day 1, (**B**) day 2, and (**C**) day 3, demonstrating that rapid uptake of ABT-806i in known glioblastoma (arrow) is identified as early as day 2 and increases over time. (**D**) SPECT/MR images showing high uptake of ABT-806i in glioblastoma (arrow) in a posterior fossa lesion. Visualization of ABT-806i uptake in the anterior venous sinus is due to blood-pool activity. Reprinted with permission: Gan, H.K., et al. A Phase 1 and Biodistribution Study of ABT-806i, an ^111^In-Radiolabeled Conjugate of the Tumor-Specific Anti-EGFR Antibody ABT-806. J Nucl Med, 2021. 62 (6): p. 787–794 [20].

The novel anti-EGFR mAb, 806 (mAb806) targets a tumor-selective epitope of EGFR that is exposed on overexpressed, mutant, or ligand-activated forms of EGFR. Significantly, mAb806 does not bind to EGFR in normal tissues [16,17]. In a phase I study, ^111^In-labeled ch806, a chimeric form of mAb806, demonstrated excellent tumor uptake in a variety of solid tumors, and with no normal tissue uptake [18,19]. The humanized form of mAb806 (ABT-806) lacks conventional anti-EGFR mAb toxicities and is well tolerated [17]. Furthermore, biodistribution studies of ^111^In-ABT-806 (ABT-806i) showed high uptake in tumor, and no binding to normal tissue expressed EGFR, thus confirming the tumor-specific nature of mAb806 (Figure 1) [20].

### 2.2. HER2

Given the predictive and prognostic value of HER2, in particular, in breast and gastric cancers, and the heterogeneity of expression of HER2 in some tumors, accurate determination of HER2 status is critical [21]. Numerous preclinical studies have shown high receptor saturation and tumor uptake of radiolabeled pertuzumab and trastuzumab, using a variety of radionuclides, including zirconium-89, copper-64, iodine-131, lutetium-177, and indium-111 [22,23,24,25,26,27]. In a biodistribution study, ^89^Zr-trastuzumab demonstrated high and HER2-specific tumor uptake; furthermore, previously undetected brain metastases were identified [28,29]. Other studies have shown the potential of imaging HER2 with ^89^Zr-trastuzumab to determine HER2 status and tumor heterogeneity and to predict response to anti-HER2 therapies [30,31,32]. While most of the studies have been performed in breast cancer, a study of ^89^Zr-trastuzumab in esophagogastric cancer patients demonstrated high HER2-specific tumor uptake [33]. Apart from ^89^Zr, trastuzumab has also been radiolabeled with various radionuclides; ^64^Cu-labeled trastuzumab has comparable tumor-to-tissue ratios to ^89^Zr-trastuzumab and is able to identify primary and metastatic lesions with low uptake seen in normal tissue, with the average tumor to non-tumor ratio for primary tumors and metastatic lesions calculated to be 3.1 and 3.2, respectively [34,35,36]. Similar specific uptake was also reported in a phase I study of ^177^Lu-trastuzumab in HER2-positive primary and metastatic breast lesions [37]. ^89^Zr-pertuzumab has similarly shown to successfully identify HER2-positive disease in patients with HER2-positive andHER2-negative metastatic breast cancer [38,39]. Despite their specificity and affinity for HER2, radiolabeled-trastuzumab and pertuzumab reveal high liver uptake due to catabolism of the mAb-chelate which impacts on the detection of liver metastases [34,37]. 

### 2.3. CAIX

**Figure 2:**
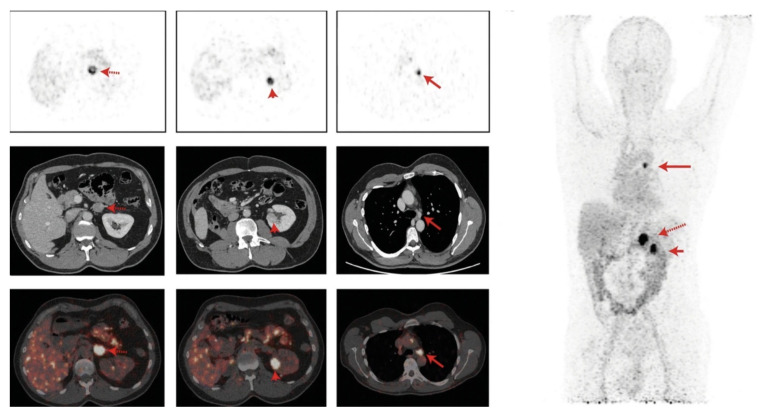
^89^Zr-girentuximab in renal cancer patient. Left panel: top row, axial PET imaging at 168 h post administration (h p.a).; middle row, contrast enhanced CT; bottom row, fused PET/CT imaging showing (from left to right) ^89^Zr-girentuximab uptake in the adrenal gland (red arrow), left kidney (red arrowhead), and mediastinal lymph node (red arrow). Right panel: the maximum intensity projection (MIP) whole-body PET image at 168 h p.a. Reprinted with permission from Merkx, R.I.J.; Lobeek, D.; Konijnenberg, M.; et al. Phase I study to assess safety, biodistribution, and radiation dosimetry for ^89^Zr-girentuximab in patients with renal cell carcinoma. Eur J Nucl Med Mol Imaging, 2021. 48, 3277–3285 [43].

Carbonic anhydrase IX (CAIX) is a hypoxia-induced enzyme expressed on cancer cells and associated with treatment resistance [40]. CAIX is overexpressed in clear-cell renal-cell tumors (ccRCC) due to a mutation in the Von Hippel–Lindau protein [41]. A number of biodistribution studies have evaluated ^89^Zr-labeled girentuximab, an anti-CAIX monoclonal antibody [42,43,44]. These studies have shown ^89^Zr-girentuximab was safe and allowed differentiation between ccRCC and non-ccRCC lesions, enabling early detection of tumor recurrence and inform clinical decision making (Figure 2). In a phase III study, the imaging of CAIX with ^124^I-girentuximab was shown to accurately identify ccRCC lesions with the diagnostic equivalence to biopsy [45]. A phase III study (ZIRCON) is underway, assessing the sensitivity and specificity of ^89^Zr-girentuximab in detecting ccRCC (NCT03849118).

### 2.4. Mesothelin

Mesothelin (MSLN) is a cell-surface glycoprotein that is highly expressed in several tumor types, including pancreatic cancer, ovarian cancer, and malignant pleural mesothelioma [46,47]. The biological function of MSLN is not fully understood. A variety of MSLN-targeting agents, including mAbs (e.g., EPR4509, EPR19025-42, MMOT0530A, and amatuximab), ADCs (anetumab ravtansine), immunotoxin (SSP1), and CAR-T cells have been conjugated to tracers and tested in malignancies overexpressing MSLN to determine mesothelin expression and correlate with tumor uptake and response to treatment [47]. All the tracers showed specific tumor uptake and the potential to predict the response to MSLN-directed therapies [47]. In addition, tumor uptake was MSLN-mediated and correlated with antibody dose and tumor size [48,49]. In a phase I study, the anti-MSLN antibody MMOT0530A radiolabeled with ^89^Zr- [50] demonstrated maximum tumor uptake four days’ post-injection (mean SUVmax of 13.1 (±7.5)), with uptake observed in at least one tumor lesion in all patients (range 1–8). Heterogeneity of ^89^Zr-MMOT0530A tumor uptake occurred both intra-patient, with a 2.4-fold mean tumor uptake difference, and inter-patient, with a mean difference of 5.3-fold. The tracer tumor uptake (SUVmax on day 4) correlated with MSLN expression, as determined by immunohistochemistry (IHC) on archival tumor tissue [50]. 

### 2.5. Programed Death 1 (PD-1) and Programmed Death-Ligand 1 (PD-L1)

Immune checkpoint inhibitors, in particular, anti-PD1 and anti-PDL1 therapies, have changed the treatment paradigms in a number of tumor types, demonstrating objective and durable responses. PD-L1 expression testing by IHC is the most widely used biomarker of response to immunotherapy, despite being predictive of response in less than a third of cases. Furthermore, multiple studies have shown the efficacy of immunotherapies in patients with PD-L1-negative tumors [51]. A number of factors limit the predictive value of PDL-1 expression as determined by IHC, including the heterogeneity of PD-L1 expression, differences in PD-L1 scoring systems, staining platforms, and the types of cells tested for PD-L1 expression, as well as the dynamic changes in PD-L1 expression over time. Non-invasive molecular imaging of PD-(L)1 expression, using radiolabeled tracers, is being evaluated in clinical trials to overcome these limitations and is supported by a number of preclinical studies [52,53,54,55,56,57,58]. Preclinical studies show tracer specificity for PD-L1 irrespective of the radionuclide (indium-111, copper-64, and zirconium-89), with uptake correlating with levels of PD-L1 expression, allowing for the assessment of inter- and intra-tumoral heterogeneity [59]. Clinical studies have shown variable uptake in tumors that better correlated with clinical responses than IHC or RNA-sequencing-based approaches [60,61]. However, significant tracer uptake heterogeneity between patients and between different lesions within the same patient, high radiotracers accumulation in the spleen and liver, and poor central nervous system (CNS) tracer penetration are significant challenges that still need addressing [61]. 

## 3. Radiolabeled Antibodies for Cancer Treatment

When a radiolabeled mAb binds to its target antigen, the emitting radionuclide results in DNA-strand breaks through the generation of free radicals, resulting in apoptosis and programmed necrosis. In contrast to other anticancer therapies, radioimmunoconjugates may not require internalization (with the exception of alpha-particle therapy) and do not target specific signaling pathways to exert their antitumor effects. Compared to naked mAbs, treatment with radiolabeled mAbs can result in high response rates with lower toxicities, particularly when compared to many other systemic cancer treatments [62]. However, toxicity due to red marrow exposure related to the circulating half-life of intact mAbs is generally dose limiting. For therapeutic purposes, most radioimmunoconjugates contain either alpha-particle- or beta-particle-emitting radionuclides. The β-emitters are light energetic electrons that travel over a relatively long path length (0.5–12 mm) and have a low LET (approximately 0.2 keV/μm). They induce the formation of free radicals, causing DNA damage [63]. Examples of β-emitting radionuclides include iodine-131, copper-67, lutetium-177, or yttrium-90; iodine-131 and yttrium-90 have been used in most clinical RIT trials and are considered the current standard to which all other therapeutic radionuclides are compared [64]. In contrast, α emitters are positively charged heavy particles with a short-range path length of (20–100 μm) and high LET (approximately 100 keV/μm) [65].

### 3.1. Approved Radioimmunoconjugates

^131^I-tositumomab (Bexxar^®^; GlaxoSmithKline) and ^90^Y-ibritumomab tiuxetan (Zevalin^®^; Biogen Idec) have been approved by the US Food and Drug Administration (FDA) and European Medicines Agency (EMA) in the management of non-Hodgkin B-cell lymphoma (NHL). ^131^I-chTNT (Cotara^®^) for the treatment of refractory advanced lung cancer and ^131^I-Metuximab (Licartin^®^) for the treatment of hepatocellular carcinoma (HCC) have been approved by the Chinese National Medical Products Administration (NMPA) [66].

^90^Y-Ibritumomab tiuxetan (Zevalin^®^; Biogen Idec) comprises the CD20 targeting mAb, ibritumomab, linked to the radionuclide yttrium-90. Patients treated with ^90^Y-Ibritumomab receive a dose of unlabeled rituximab to block CD-20+ binding sites on B-cells located in the circulation and in the spleen. The dose of ^90^Y-Ibritumomab is calculated based on the patient’s weight and platelet count. ^90^Y-Ibritumomab was first approved in 2002 for the treatment of low-grade or follicular lymphoma refractory to rituximab and relapsed or refractory low-grade follicular or transformed lymphoma. Then, in 2009, ^90^Y-Ibritumomab received expanded approval for treatment of patients with previously untreated follicular NHL who have achieved a partial or complete response to first-line chemotherapy. Several studies have demonstrated the superiority of ^90^Y-ibritumomab tiuxetan over rituximab. In a phase III trial, treatment with ^90^Y-ibritumomab tiuxetan resulted in superior overall response rates (ORR) (80% versus 56% for the rituximab group) and a number of complete responses (30% versus 16% in the rituximab group) [67,68,69]. In addition, the combination of ^90^Y-ibritumomab tiuxetan with chemotherapy resulted in improved progression-free survival (PFS) (2-year PFS 59% versus 39% for the chemotherapy-alone group) and overall survival (OS) (2-year OS 91% versus 62% for the chemotherapy group alone) with no significant added toxicity [70]. In a randomized phase III trial (First-Line Indolent trial), ^90^Y-ibritumomab tiuxetan was shown to be efficacious as consolidation therapy in patients with advanced-stage follicular lymphoma in first remission. After a median follow-up of 7.3 years, PFS was significantly improved with ^90^Y-ibritumomab tiuxetan (41% versus 22% for control group), with a prolonged median time to next treatment (8.1 years versus 3.0 years for the control group) [71,72]. Similar benefits of ibritumomab tiuxetan as consolidation therapy have been shown in other studies in patients with intermediate and high-risk follicular lymphoma [73,74,75]. 

Tositumomab and ^131^I-tositumomab (Bexxar^®^; GlaxoSmithKline), an anti-CD20 mAb regimen, received FDA approval in June 2003 for the treatment of CD20-positive relapsed/refractory follicular NHL. Similar to ^90^Y-Ibritumomab, prior to administration of ^131^I-tositumomab, unlabeled tositumomab is administered to bind B-cells in circulation and in the spleen. A meta-analysis of heavily pretreated patients (received at least four prior regimens) with indolent lymphoma treated with ^131^I-tositumomab reported ORR of 68% [76]. Treatment-naive patients treated with chemotherapy, followed by one dose of tositumomab and ^131^I-tositumomab, had a 100% response rate with the median response duration not reached after a median follow-up of 8.4 years [77]. There was, however, no difference in PFS (2-year PFS 48.6% versus 47.9%, respectively) or OS (2-year OS 65.6% versus 61%, respectively) reported in a phase III trial comparing rituximab plus chemotherapy with ^131^I-tositumomab and chemotherapy with autologous hematopoietic cell transplantation for relapsed diffuse large B-cell lymphoma [78]. RIT consolidation in patients with follicular lymphoma has shown encouraging results in a meta-analysis; however, further studies comparing the benefits of consolidation RIT to maintenance rituximab are required [79]. Tositumomab and ^131^I-tositumomab were withdrawn from the market in 2014, due to lack of commercial demand [1]. 

^131^I-chTNT has been approved by the NMPA in China for the treatment of refractory advanced lung cancer [66,80]. Tumor necrosis treatment (TNT) differs from other ACTs through the targeting of necrotic regions within the tumor rather than surface antigens [81]. Based on promising efficacy data, ^131^I-chTNT has received approval from the Chinese State Food and Drug Administration for the treatment of patients with advanced lung cancer who were previously treated with radiotherapy or chemotherapy [82]. In the pivotal study, pretreated patients with advanced lung cancer received intravenous, intra-tumoral or a combination of intravenous and intratumoral ^131^I-chTNT. The ORR was 34.6%; patients receiving intratumoral injection only (*n* = 16) had an ORR 56%, and patients treated with a combination of intravenous and intratumoral (*n* = 5) had an ORR of 40% [80]. Response rates were similar irrespective of route of administration. Hematological toxicity was most commonly reported in patients who received RIT intravenously. 

^131^I-metuximab (Licartin^®^, Chengdu Huashen Biotechnology) is a radioimmunoconjugate targeting CD147, which is a transmembrane glycoprotein associated with hepatocarcinogenesis, HCC growth, and metastasis [83]. The combination of ^131^I-metuximab with transcatheter arterial chemoembolization or radiofrequency ablation in patients with intermediate and advanced HCC has shown be safe and resulted in delayed recurrence and improved OS [84,85,86,87]. In addition, in a randomized trial, ^131^I-metuximab post-orthotopic liver transplantation significantly reduced rates of recurrence rates (by 30.4%) and increased survival (by 20.6%) versus placebo [88]. ^131^I-metuximab is approved by the China State Food and Drug Administration for the treatment of primary HCC.

### 3.2. Radioimmunoconjugates in Development

#### 3.2.1. Solid Tumors

In contrast to hematological malignancies, the efficacy of radiolabeled mAbs in solid tumors has been modest, with responses seen mainly in tumors with high antigen expression, and those treated with fractionated RIT protocols or with the combination of RIT with other agents, typically chemotherapy [89]. Efficacy of RIT in solid tumors is limited by factors not present in hematological tumors, such as poor perfusion and heterogeneities in blood flow the presence of tumor stroma, heterogeneous target antigen expression, and inherent resistant to radiation therapy. New approaches, including fractionated RIT, modified pharmacokinetics, alpha-particle labeled antibodies, and combination with radiation sensitizers, may provide enhanced efficacy and are being evaluated in clinical trials.

##### Renal Cell Carcinoma

In a phase I study of ^177^Lu-girentuximab in patients with metastatic ccRCC [90] myelotoxicity was dose limiting and 17/23 (74%) patients showed stable disease three months after the first treatment, with just over a half (54%) receiving a second dose. In a follow-up phase II study of 14 patients, eight patients (57%) were found to have stable disease and one (7%) had a partial regression after the first treatment. Myelotoxicity was observed in most patients, and it prevented retreatment in some patients [91]. 

##### Prostate Cancer

Prostate-specific membrane antigen (PSMA) is a transmembrane protein that is overexpressed in castrate-resistant tumors, making it an attractive theranostic target. In an early phase trial, CYT-356, a PSMA-targeting mAb, radiolabeled with ^90^Y, had no therapeutic effect, with myelosuppression being the dose-limiting toxicity (DLT) [92]. A phase II study was stopped early, due to futility [93]. 

**Figure 3:**
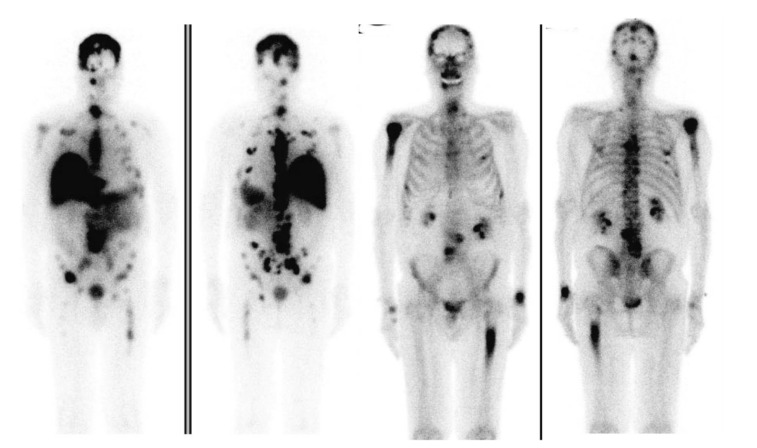
Left to right: ^177^Lu-J591 anterior and posterior whole-body images 7 days after ^177^Lu-J591 administration, and Technetium 99m-methyl diphosphonate (^99m^Tc-MDP) bone scan anterior and posterior whole-body images, showing ^177^Lu-J591 targeting of extensive bone and soft-tissue metastases. Reprinted with permission from: Tagawa S.T, et al. Phase 1/2 Study of Fractionated Dose Lutetium-177–Labeled Anti–Prostate-Specific Membrane Antigen Monoclonal Antibody J591 (^177^Lu-J591) for Metastatic Castration-Resistant Prostate Cancer. Cancer, 2019 Aug 1; 125 (15): 2561–2569 [97].

Hu-J591 (rosopatamab) mAb targeting PSMA radiolabeled with yttrium-90 and lutetium-177 via a DOTA chelate was studied in two phase I studies [94,95]. J591 demonstrated good tumor targeting in nearly all patients (42/43), and dose related antitumor activity and PSA decline were reported in both trials. A phase II study of ^177^Lu-J591 in heavily pretreated patients with prostate cancer was undertaken, confirming the safety, efficacy, and tumor-targeting ability [96]. A phase I study combining docetaxel for its radiosensitizing properties with ^177^Lu-J591, which was administered in fractionated dosing, showed that the combination was well tolerated; however, grade 4 myelosuppression was reported in 30% (5/15) of patients (Figure 3) [97]. ^177^Lu-labeled J591 therapy is now under investigation in the salvage setting (NCT00859781), as well as in the second line setting for the treatment of PSMA-expressing castrate-resistant metastatic prostate cancer (PROSTACT; NCT04876651).

PSMA-targeting radiolabeled small molecules, such ^177^Lu-PSMA-617 and ^225^Ac-PSMA-617, have demonstrated clinical and biochemical responses with low toxicity in chemotherapy-naive patients, as well as patients who have progressed on standard therapeutic options, respectively [98,99,100]. The success of these treatments has further increased interest in PSMA-directed RIT.

##### Colon Cancer 

Carcinoembryonic antigen (CEA) is a glycoprotein produced by >90% of colonic epithelial cells. CEA plays an important role as a ligand involved in cancer dissemination [101]. In a phase II study, patients with metastatic colorectal cancer who received a single dose of the radiolabeled humanized anti-CEA mAb ^131^I-labetuzumab, following R0 resection of liver metastases, had superior OS compared to a similar contemporaneous group of control patients that did not receive RIT [102,103]. The main toxicities reported were hematological. A larger phase II study using ^131^I-labetuzumab as “adjuvant” treatment in mCRC patients, post-metastectomy, resulted in a median time to progression and OS of 16 and 55 months, respectively; however, significant grade 4 hematological toxicity was seen, including myelodysplastic syndrome in 5/63 patients enrolled [104]. 

The A33 cell surface antigen is expressed in >95% of human colon cancers, and in normal colonic and small bowel epithelium. It is, however, absent in other epithelial tissues [105]. RIT trials of iodinated murine A33 mAb (^131^I-muA33 and ^125^I-muA33) showed safety, tolerability, and specific tumor-targeting in patients with advanced colorectal cancer, with modest antitumor activity [106,107]. A humanized IgG1 huA33 antibody has high affinity for A33, and in clinical trials, it has been shown to have an acceptable toxicity profile [108]. A biodistribution study of ^131^I and ^125^I labeled huA33 showed excellent tumor uptake and penetrated to the center of large necrotic metastatic lesions. No dose-limiting toxicities were reported [109]. In a phase I trial of ^131^I-huA33 in pretreated patients with mCRC, hematological toxicity was seen to be dose dependent and dose limiting. Excellent tumor localization was seen, along with some antitumor effects [110]. ^131^I-huA33 in combination with capecitabine resulted in greater tumor responses than those observed with ^131^I-huA33 alone with no significant added toxicity [111]. 

In a dose-escalation study, the ^125^I-radiolabeled chimeric antibody, 17-1A, directed against epithelial cell-adhesion molecule (Ep-CAM), no dose-limiting toxicities were observed, albeit no tumor responses [112].

Panitumumab conjugated to α-emitter, ^212^Pb, resulted in improved survival in a preclinical CRC model, with enhanced benefit observed when combined with chemotherapy [113].

##### Ovarian Cancer

RIT in ovarian cancer is typically administered intraperitoneally rather than by intravenous administration. Responses typically correlate to tumor volume, with higher responses observed in patients with minimal residual disease or with low disease burden [114].

MUC1, a member of the mucin family, is a glycosylated protein that is overexpressed in a variety of epithelial cancers. MUC1 plays a crucial role in tumorigenesis, cancer progression, and treatment resistance [115]. In a phase II trial, patients with ovarian cancer in remission after chemotherapy treated with ^90^Y labeled anti-MUC1 antibody, HMFG-1, had a significant improvement in five-year survival compared to the controls (80% vs. 55%, respectively) [116]. In a randomized phase III multicenter trial, the addition of RIT resulted in a decrease in intraperitoneal relapse rates, but this did not translate to an improvement in OS when compared with standard care [117,118].

Tumor-associated glycoprotein 72 (TAG-72) is overexpressed in a range of solid tumors. CC49, an anti-TAG-72 antibody, has shown excellent tumor targeting, albeit minimal antitumor activity as an unlabeled antibody [119]. A number of anti-TAG-72 intact antibodies radiolabeled with iodine-131, yttrium-90, and lutetium-177 have been evaluated clinically [120,121,122]. In an early phase study, patients with treatment-resistant recurrent/persistent ovarian cancer received ^90^Y-CC49 in combination with intraperitoneal paclitaxel and interferon. Of the patients with measurable disease, two had PRs lasting 2 and 4 months, while 11 patients with non-measurable disease had a median time to progression of 6 months [122]. CC49 radiolabeled with alpha-emitters have also been tested in preclinical ovarian cancer models with promising results [123].

A phase I multicenter trial of ^225^Ac-FPI-1434 (anti-IGF-1R mAb) is currently being conducted in patients with solid tumors that show uptake of ^111^In-FPI-1547 (NCT03746431). Several other potential targets and radiolabeled antibodies have been evaluated in patients with solid tumors, showing acceptable safety profiles but modest antitumor activity [124].

##### Pancreatic Ductal Adenocarcinoma (PDAC) Cancer

Clinical trials investigating RIT in PDAC have predominantly used β-emitting radionuclides, and, to our knowledge, α RIT has not been tested in a clinical trial in PDAC. The most common target investigated is mucin 1 (MUC1), which is overexpressed in >60% of PDAC. However, the exact role of MUC1 in PDAC is still unknown [125]. In a phase I trial, ^90^Y-clivatuzumab tetraxetan was well tolerated with manageable hematologic toxicity [126]. The addition of low-dose gemcitabine resulted in improved disease control and OS [127,128]. However, a phase III study (PANCRIT^®^-1) investigating the combination was prematurely terminated due to futility after an interim analysis (NCT 01956812).

Over 90% of pancreatic cancers overexpress CEA [129]. In a phase I/II study KAb201, an anti-CEA mAb that was radiolabeled with isodine-131 was well tolerated, but it had only modest efficacy. ^131^I-KAb201 was administered either intravenously or intra-arterially, with no significant difference in therapeutic efficacy seen between the administration routes [130].

##### Primary Brain Tumors

Tenascin is a multi-domain extracellular matrix glycoprotein that is highly expressed in several cancers, including in high-grade gliomas [131]. ^131^I-labeled mAb 81C6, a tenascin-targeting mAb, demonstrated high specificity in biodistribution studies [132]. These studies also showed limited intra-tumoral penetration following intravenous or intra-arterial administration, and subsequent studies have used intracranial administration [133]. A number of dosimetry and phase I/II studies have investigated ^131^I-81C6 in patients with newly diagnosed malignant high-grade gliomas. These studies used a single dosing regimen and demonstrated improvements in survival rates compared to historical controls and significantly minimal hematologic and neurological toxicity, including lower rates of radionecrosis. A correlation between the radiation-absorbed dose and tumor recurrence or radionecrosis was also reported [134,135,136,137,138,139]. Other studies demonstrated improvements in survival rates for patients with newly diagnosed and recurrent glioblastoma treated with repeated doses of ^131^I-labeled anti-tenascin murine antibodies, BC-2 and BC-4 [140,141,142]. Moreover, 81C6 mAb has also been radiolabeled with astatine-211, an alpha emitter, and tested in patients with recurrent gliomas [133]. No dose-limiting toxicities were reported, and there was an improvement in median survival times (54 weeks versus 23–31 weeks for patients treated with conventional therapies). 

EGFR aberrations, including amplification, deletion, or mutation, are detected in ~60% of glioblastoma [143]. Two large phase II studies evaluated an EGFR targeting mAb radiolabeled with iodine-125, ^125^I-mAb 425, in patients with high-grade gliomas. These studies demonstrated that RIT was well tolerated and resulted in a survival benefit compared to a contemporaneous control group that did not receive RIT [144,145]. Another EGFR RIT-targeting modality, nimotuzumab radiolabeled with rhenium-188, was evaluated in a phase 1 study in patients with high-grade glioma as a single-dose intracavitary injection. Durable responses were observed in three patients, lasting more than one year; however, early severe neurological symptoms and late-onset radionecrosis were observed with the higher dose of ^188^Re [146]. Other potential RIT targets showing promise in gliomas in preclinical studies include carbonic anhydrase, cadherin 5, and Integrin αVβ3 [147]. 

### 3.3. Hematological Cancers

#### 3.3.1. Lymphoma

Several RIT agents targeting populations of cells expressing CD21, CD22, CD37, and the human leukocyte antigen DR (HLA-DR) are being investigated. 

CD22 is a transmembrane glycoprotein that is widely expressed across malignant B-cell histologies [148]. In contrast to Zevalin^®^ and Bexxar^®^, the anti-CD22 ^90^Y-epratuzumab tetraxetan can be administered without a loading dose of cold antibody. A phase I/II trial assessed ^90^Y-epratuzumab in patients with relapsing B lymphoma [149]. Patients had imaging studies one week prior to treatment with ^111^In-epratuzumab. The trial enrolled patients in two cohorts: patients who had prior high-dose chemotherapy (Group 2) and patients who did not have a prior stem-cell transplantation (SCT) (Group 1) [149]. Irrespective of tumor targeting and tumor size, antitumor responses were seen in both indolent and aggressive NHL. Similarly, antitumor responses have also been seen from using fractionated dosing of ^90^Y-epratuzumab with toxicities that were primarily hematological and dose dependent [150,151]. The efficacy of fractionated ^90^Y-epratuzumab as consolidation therapy after rituximab-based therapy was investigated in elderly (age>60 years) patients presenting with stage I/II bulky or stage III/IV diffuse large B-cell lymphoma (DLBCL). The estimated 2-year event-free survival was 75%, and grade 3/4 thrombocytopenia and neutropenia were seen in 84% and 79% patients, respectively. One patient each developed myelodysplastic syndrome and acute myeloid leukemia post-RIT [152,153]. 

BAY 1862864 is a CD22-targeting antibody radiolabeled to the α-particle emitting radionuclide thorium-227. BAY1862864 was evaluated in a first-in-human study in patients with CD22-positive relapsed/refractory B cell NHL [154]. In this study, the DLTs were febrile neutropenia and thrombocytopenia, with nearly half the patients (48%) developing grade ≥3 treatment-emergent adverse events, with the most common being myelotoxicity. The MTD was not reached. The ORR reported was 25%, with responses higher in patients with relapsed low-grade versus high-grade lymphomas (30% vs. 11%, respectively).

Lym-1, a monoclonal antibody which preferentially targets malignant B-lymphocytes, when labeled with iodine-131, has been shown to induce durable remissions in patients with NHL resistant to chemotherapy [155,156]. Dose-limiting toxicity was thrombocytopenia, and non-hematologic toxicities were typically of low grade. Dose-limiting toxicity of ^90^Y-Lym-1 was also thrombocytopenia and more than half of the patients enrolled onto the study had a partial response or stabilization of chemotherapy-refractory NHL after RIT [157]. 

Other antigens have been investigated as targets for RIT in lymphoma in early phase studies, including CD21 [158,159] and CD37 [160,161,162]. 

#### 3.3.2. Multiple Myeloma

Numerous cell-surface antigens expressed on multiple myeloma (MM) cells have been targeted by mAbs, including anti-CD20 rituximab, anti-CD33, anti-CD38, anti-CD54, anti-CD74, anti-CD317, and anti-CD319 [2]. MM cells display inherent sensitivity to radiation, providing strong rationale to extend the use of mAbs to RIT. 

CD33 antigen is expressed on MM plasmocytes in 20–35% of MM patients and is associated with unfavorable cytogenetics and a poor prognosis [163,164]. ^225^Ac-lintuzumab is a CD33-directed RIT. A phase I study of ^225^Ac-lintuzumab in patients with MM that have failed standard therapy is underway (NCT02998047) [165]. 

CD38 is highly expressed on MM plasmocytes’ cell surface (>90%) and has immunomodulatory effects [166]. ^211^At-OKT10-B10 (^211^At-CD38) is an anti-CD38 mAb radiolabeled with the alpha-emitting radionuclide astatine-211 [167]. Preclinical studies showed excellent tumor-to-normal-organ ratios, and only modest responses were seen in a bulky xenograft model of MM. However, in a disseminated disease model designed to reflect low-burden minimal residual disease, RIT produced sustained remission [167]. A phase I clinical trial evaluating ^211^At-OKT10-B10 in combination with an ASCT-conditioning regimen in MM patients is underway (NCT04466475). Another CD38 targeting radioimmunoconjugate demonstrated prolonged tumor retention and potent antitumor effects compared to the naked antibody, without any significant toxicity [168].

CD20 is heterogeneously expressed on myeloma cells with the presence of clonogenic CD20-positive precursor B cells in MM seen in 13–22% of patients [164,169]. In a single center study, ^90^Y-Ibritumomab tiuxetan was evaluated in combination with high-dose melphalan prior to ASCT in MM patients [170]. Despite reporting encouraging results, a subsequent phase II trial was prematurely terminated due to modest response rates and increase in peri-ASCT bacterial infections [171]. 

Syndecan-1 (CD138), a transmembrane proteoglycan, is highly expressed on MM cells [172]. In a phase I/II bio-imaging trial, a single therapeutic dose of ^131^I-B-B4 showed a high uptake in the bone marrow and liver, with all patients experiencing transient grade 3 or higher hematologic toxicities. No objective responses were observed [173]. 

#### 3.3.3. Acute Myeloid Leukemia

Acute Myeloid Leukemia (AML) cells are characterized by aberrant expression of myeloid and lymphoid lineage markers. CD45 is a transmembrane glycoprotein highly expressed on all hematopoietic cells and most AML cells; therefore, it is ideal to target the bone marrow [174]. In an early phase study, ^131^I-BC8, a radiolabeled anti-CD45 mAb, in combination with cyclophosphamide and 12-Gy total body irradiation (TBI) in patients with refractory AML/ALL demonstrated favorable biodistribution, with a higher estimated radiation absorbed dose to marrow and spleen than to normal organs. The overall leukemia-free survival (LFS) was 29% [175]. Subsequently, two phase II studies were undertaken in patients with AML in a first remission (CR1) and in patients with advanced AML [176,177]. Patients with advanced AML, matched related (MRD) and unrelated (MUD) donors, were treated with TBI 12Gy with chemotherapy and ^131^I-BC8. The LFS was 42% with outcomes identical in the MRD and the MUD groups [176]. Patients with AML in CR1 with intermediate-risk or high-risk cytogenetic features received RIT in combination with chemotherapy, using family donors. Overall LFS was 60% with the LFS higher for intermediate-risk vs. high-risk patients (68 vs. 40%, respectively) [177]. 

CD33 is only expressed on myeloid cells and lymphocytes and frequently expressed on AML cells [178]. Lintuzumab (SGN-33 and HuM195) is CDR-grafted IgG1 humanized mAb directed against CD33 (179). In a phase I trial, Lintuzumab showed similar pharmacology to murine M195 and was well-tolerated without significant immunogenicity [179]. ^131^I-Lintuzumab in combination with busulfan and cyclophosphamide as a conditioning regimen for allogeneic bone-marrow transplantation was well tolerated and resulted in prolonged myelosuppression. A fifth of patients with relapsed/refractory AML in this study experienced significant improvements in long-term survival [180]. In an attempt to avoid the myelosuppression experienced with β-emitting constructs, the α-emitters bismuth-213 and actinium-225 were conjugated to lintuzumab. Both radioconjugates have demonstrated encouraging results in phase I/II in AML patients [181,182]. Based on this, a number of trials are evaluating ^225^Ac-lintuzumab in AML patients (NCT03867682, NCT03932318, NCT02575963, and NCT03441048) and in patients with CD33-positive multiple myeloma (NCT02998047).

## 4. Conclusions

Radioimmunoconjugates have been shown to be useful for both the detection of tumors and for therapy, and can be combined with conventional therapies to enhance the therapeutic efficacy of mAbs [114]. In addition, the use of mAb radioconjugates as imaging theranostics allows for real-time antigen quantitation, detect heterogeneity, and dynamic changes in antigen expression, all of which can be vital for guiding and monitoring therapy responses, as well as in drug development, providing important information concerning pharmacokinetics of mAbs and patient selection and drug dose required for therapeutic efficacy. Despite RIT demonstrating therapeutic efficacy in hematological malignancies, the same benefit has yet to be seen in solid tumors and represents the principal challenge of the future. Several strategies have been proposed to improve their therapeutic index and are being evaluated, including targeting patients with minimal disease burden, using pre-targeting strategies, newer radionuclides (including alpha-particle isotopes), and using combined therapeutic modalities (such as DNA sensitizers) and locoregional application [114]. Furthermore, improvements in radiochemistry and scale of production may help to reduce the high production costs and allow for the wider use of these therapies. The opportunities for RIT are expanding, and further trials will demonstrate the potential of this approach in treating cancer patients.

## Figures and Tables

**Table 1:** Examples of clinical trials evaluating radiolabeled antibodies for imaging and therapy.

Antigen	Radiolabeled Antibody	Application	Tumor	Phase	Trial Status	References
PD-L1 and PD1	^89^Zr-atezolizumab	Diagnostic	Breast cancer	Pilot	Recruiting	NCT04222426
	^89^Zr-atezolizumab	Diagnostic	Renal cell carcinoma	I	Recruiting	NCT04006522
	^89^Zr-durvalumab	Diagnostic	Lymphoma	I	Recruiting	NCT03610061
	^18^F-PDL1	Diagnostic	Lung cancer	Pilot	Recruiting	NCT03564197
	^18^F- atezolizumab	Diagnostic	Esophageal and rectal cancer	Pilot	Active, not recruiting	NCT04564482
	^89^Zr-envafolimab (KN035)	Diagnostic	PD-L1 positive solid tumors	Pilot	Recruiting	NCT04977128
	^89^Zr-atezolizumab	Diagnostic	Lymphoma	Pilot	Recruiting	NCT03850028
	^68^Ga-WL12	Diagnostic	Gastrointestinal tumors	Pilot	Recruiting	NCT04629326
	^89^Zr-M7824	Diagnostic	NSCLC	1	Recruiting	NCT04297748
	^89^Zr-MPDL3280A	Diagnostic	Solid tumor	Pilot	Recruiting	NCT02453984
	^89^Zr-atezolizumab	Diagnostic	RCC	1	Recruiting	NCT04006522
	^89^Zr-REGN3504	Diagnostic	HCC and Gastric/GEJ tumors	1	Recruiting	NCT03746704
	^89^Zr-pembrolizumab	Diagnostic	NSCLC	2	Status unknown	NCT03065764 ^±^
	^89^Zr-crefmirlimab	Diagnostic	Melanoma, MCC, RCC, NSCLC	2	Recruiting	NCT05013099
	^64^Cu-pembrolizumab	Diagnostic	Hematological and solid tumors	1	Recruiting	NCT04605614
PSMA	^177^Lu-HuJ591 + Ketoconazole	Therapeutic	Prostate cancer	II	Recruiting	NCT00859781
	^177^Lu-7E11-C5.3	Therapeutic	Prostate cancer	I	Status unknown	NCT00441571 ^±^
CAIX/MN	^89^Zr-girentuximab	Diagnostic	Urothelial cancers	I/II	Recruiting	NCT05018442
	^89^Zr-girentuximab	Diagnostic	Clear cell renal cell cancer—(ZIRCON)	III	Recruiting	NCT03849118
	^89^Zr-girentuximab	Diagnostic	Non-muscle invasive bladder cancer	I	Recruiting	NCT04897763
	^89^Zr-girentuximab	Diagnostic	Urothelial cancers	I	Recruiting	NCT05046665
	^89^Zr-girentuximab	Diagnostic	Triple negative breast cancer	II	Recruiting	NCT04758780
IGF-1R	^225^Ac-FPI-1434	Therapeutic	IGF-1R expressing solid tumors	I/II	Recruiting	NCT03746431
GD2	^131^I-3F8	Therapeutic	Brain tumors and leptomeningeal disease	II	Active, not recruiting	NCT00445965
EGFR	^125^I-425	Therapeutic	Brain tumors	I	Status unknown	NCT01317888 ^±^
	^89^Zr-Nimotuzumab	Diagnostic	Lung and colorectal cancers	I/II	Recruiting	NCT04235114
	^89^Zr-Panitumumab	Diagnostic	Colorectal cancers	I/II	Recruiting	NCT03764137
	^89^Zr-ABT806	Diagnostic	High grade glioma	Pilot	Status unknown	NCT03058198
HER2	^64^Cu-Trastuzumab	Diagnostic	Stage III Breast cancer	II	Active, not recruiting	NCT02827877
	^64^Cu-Trastuzumab	Diagnostic	Breast cancer	Pilot	Active, not recruiting	NCT01093612
	^64^Cu-Trastuzumab	Diagnostic	Breast cancer	Piot	Active not recruiting	NCT02226276
	^89^Zr-Pertuzumab	Diagnostic	HER2-Positive Solid Tumors	I	Recruiting	NCT04692831
CD25	^90^Y-basiliximab + BEAM protocol (carmustine etoposide cytarabine melphalan)	Therapeutic	HL	I	Active, not recruiting	NCT01476839
CD33	^225^Ac-Lintuzumab	Therapeutic	MM	I	Status unknown	NCT02998047
	^225^Ac-Lintuzumab + Venetoclax	Therapeutic	AML	I	Recruiting	NCT03867682
	^225^Ac-Lintuzumab + Venetoclax + Azacitidine	Therapeutic	AML	I/II	Not yet recruiting	NCT03932318
	^225^Ac-Lintuzumab	Therapeutic	AML (older patients ≥ 60 yrs)	I/II	Active, not recruiting	NCT02575963
	^225^Ac-Lintuzumab + Cladribine + Cytarabine + Filgastrim + Mitoxantrone (CLAG-M)	Therapeutic	AML	I	Recruiting	NCT03441048

Abbreviations: AML, acute myeloid leukemia; GEJ, gastro-esophageal cancer; HCC, hepatocellular carcinoma; HL, Hodgkin’s lymphoma; IGF-1R, type I insulin-like growth factor receptor; MCC, Merkel cell carcinoma; MM, multiple myeloma; NSCLC, non-small-cell lung cancer; PDL-1, programmed death ligand-1; RCC, renal cell carcinoma. ^±^ Status unknown or withdrawn (no subjects enrolled).

## Data Availability

Not applicable.
